# You Eat How You Think: A Review on the Impact of Cognitive Styles on Food Perception and Behavior

**DOI:** 10.3390/foods11131886

**Published:** 2022-06-25

**Authors:** Thadeus L. Beekman, Philip Glen Crandall, Han-Seok Seo

**Affiliations:** Department of Food Science, University of Arkansas, 2650 North Young Avenue, Fayetteville, AR 72704, USA; tlbeekma@uark.edu (T.L.B.); crandal@uark.edu (P.G.C.)

**Keywords:** analytic, behavior, cognitive, food, holistic, perception

## Abstract

Sensory perception is understood to be a complex area of research that requires investigations from a variety of different perspectives. Although researchers have tried to better understand consumers’ perception of food, one area that has been minimally explored is how psychological cognitive theories can help them explain consumer perceptions, behaviors, and decisions in food-related experiences. The concept of cognitive styles has existed for nearly a century, with the majority of cognitive style theories existing along a continuum with two bookends. Some of the more common theories such as individualist-collectivist, left-brain-right-brain, and convergent-divergent theories each offered their own unique insight into better understanding consumer behavior. However, these theories often focused only on niche applications or on specific aspects of cognition. More recently, the analytic-holistic cognitive style theory was developed to encompass many of these prior theoretical components and apply them to more general cognitive tendencies of individuals. Through applying the analytic-holistic theory and focusing on modern cultural psychology work, this review may allow researchers to be able to answer one of the paramount questions of sensory and consumer sciences: how and why do consumers perceive and respond to food stimuli the way that they do?

## 1. Introduction

Sensory science itself is a synthesis of supporting disciplines of study that relies on a cohesive blend of a range of individual research topics. These areas include, but are not limited to, food science, psychology, marketing, statistics, neuroscience, and human physiology. A common goal is to understand how and why consumers perceive sensory stimuli the way they do and then how consumers react to these stimuli. Three general segments of literature that have been able to attempt to answer this universal question of human perception and behavior include physiological, psychological, and environmental factors [1,2,3,4,5]. There is an expanding number of studies investigating and discussing the effects of the diversity of environmental and physiological factors influencing how individuals perceive and respond to food stimuli under varying conditions [4,5,6,7,8]. In comparison, there are relatively fewer peer-reviewed journal articles seeking to explain the impact of psychological variables on how consumers perceive their food. While there have been ample amounts of research into individual differences in specific psychological variables, such as food-evoked emotions [9] or personality traits [10,11] in food-related contexts, there is a research gap when looking at how psychological theories of cognitive styles apply to both group and individual differences. These cognitive style theories can encompass many of the more minute psychological variables (e.g., emotional states or responses) and thus offer a great deal of interest into understanding how consumers perceive and behave toward foods. 

Through this review, the concepts behind cognitive styles were detailed, followed by more in-depth explorations of specific cognitive styles, including a discussion of a more modern cognitive theory offering a more comprehensive understanding of sensory perception and consumer behavior towards foods. Relationships between the discussed cognitive theories and consumers’ food perception were then explored. Research gaps and opportunities were then detailed into how cognitive styles, notably the analytic-holistic theory, can help researchers better understand the ubiquitous question of how and why food is perceived the way it is by consumers. It is important to note that this review was not intended to be fully comprehensive or exhaustive of all applicable research due to the enormous size of the literature connecting sensory perception, cognitive style theories, and their potential areas of overlap. Rather, this review covers a wide breadth of topics while still diving to reasonable depths within each area of research with the intent of providing a clearer understanding of how cognitive style theories, with an emphasis on modern cultural psychology, can help to better understand consumer-food relationships. 

## 2. Cognitive Style’s Effects on Perception and Liking of Sensory Stimuli

### 2.1. Concept of Cognitive Style

Aspects of cultural psychology gained popularity in the later decades of the twentieth century, and prior mainstream beliefs that explained consumer preferences through adults having a common hardware, regardless of personal differences, were questioned [12,13,14]. As the ideals of cultural psychology were disseminated, it did not take long for them to become recognized by professionals throughout the field [15,16]. One of the more unique aspects of cultural psychology is its interdisciplinary nature [15]. Foundational to modern cultural psychology are historical examples of how philosophical and cultural differences were present among ancient societies. Historians and anthropologists have detailed how the ancient Greek and Chinese empires differed in what behaviors were encouraged within those cultures, such as Talhelm et al. [17] discussing how rice irrigation needs within Southern Chinese cultures necessitated collaborative cognitive and behavioral styles [18,19]. These historical underpinnings highlight how understanding and accounting for widespread cultural differences were important in human studies. Encompassing much of the prior research is the concept of ancient Chinese-influence in countries (Eastern) which descended from this similar cultural background produce dissimilar or contrasting results to consumers descending from ancient Greece-influenced (Western) countries. 

As cultural psychology developed, other theories and differences among people followed, most notably field (in)dependence and dialectical thinking. Field independent thinking individuals show more autonomy and articulate events as being discrete, while field dependent individuals take a wider and more connected view and rely on an increased amount of external or social contexts for their perception and decisions [20,21]. In addition, dialectical thinking is focused on decreasing conflict and finding a reasoning-based “middle way” during decision making [22,23]. By integrating these perceptual and reasoning aspects, Nisbet et al. [13] provided a more encompassing way of differentiating psychological aspects of peoples’ culture that includes field (in)dependence, dialectic thinking, and other cognitive aspects. This expanded definition discusses two ways of thinking: analytic and holistic, reflecting cultural differences in reasoning, perception, and cognition [24].

#### 2.1.1. Analytic Versus Holistic Style

Analytic and holistic categorization are manifested into two general types of cultures, with analytic and holistic thinking being more prevalent in ancient Greek-influenced (or Western) areas and ancient Chinese-influenced (or Eastern) cultures, respectively [25]. This analytic-holistic separation incorporates reasoning, perception, and cognitive processing reflective of more general cognitive styles [13]. One important caveat is that analytic and holistic are two cognitively opposite styles, yet not mutually exclusive, as findings indicate that many people capable of both styles usually have a predisposition toward one or the other [26,27]. Additional recent work also highlights how analytic and holistic groups are not equally affected by the stimuli and situations. For example, when exposed to the opposite cognitive thinking style, analytic individuals will choose the more familiar stimuli, with this effect not being seen for the holistic group [28]. This asymmetrical finding supports the fact that the groups can be differentially affected by stimuli or environmental situations. 

Analytic thinking is associated with increased attention to focal points of a situation, linearized thought, and independent interpretation of events, while holistic thinking is associated with opposite behaviors of contextual attention bias, cyclical interpretation and prediction, and interdependent relationships of events [13,14,25]. These differences between analytic and holistic cultures accentuate how each may process events or information differently. Analytic cultures tend to state that events or objects are independent and that current trends should continue in a parallel fashion [23,29]. From the holistic perspective, events and change are handled oppositely and are seen as more interrelated in the fact that there are truly no “independent” events, and change is more cyclical, repeating itself in nature [30]. Such stark differences between how analytic and holistic people process information logically produce separate cognitive styles which may lead to divergent mental categorization and decision-making strategies. 

Building upon the body of analytic-holistic research was the publication of the Analysis-Holism Scale (AHS) to measure analytic versus holistic tendencies [31,32]. Original studies on AHS development highlighted its ability to separate the traditionally holistic (Korea) and analytic (United States of America) cultures from one another, while also separating analytic and holistic cognitive groups within a single culture [32]; for additional validation of the AHS between and within cultures, see [25,33,34,35]. In addition, other researchers have recently postulated that the AHS may not be the most applicable when applied in niche applications as opposed to the more general tendencies the analytic-holistic theory was originally developed to assess [36,37,38]. While analytic and holistic cognitive styles are the focus of this review, it is still valuable to understand other types of cognitive styles that have been used to classify consumers and how they relate to analytic and holistic theory. 

#### 2.1.2. Collectivism Versus Individualism Style

Another way of categorizing individuals and their respective cognitive style is to consider their cultural belonging in a slightly different way, e.g., collectivism versus individualism. Between these two categories, there are different tendencies people within those cultures tend to display. This cognitive concept was put forth originally to help explain differences among individuals having different backgrounds and experiences [39,40]. Individualist cultures can often be formed or shaped by a multitude of smaller groups within a culture, which then induce more individualistic inclinations and less feelings of belonging among individuals [41,42]. An example of this type of culture would be the United States or Canada, in which both were formed by large influxes of various cultural groups. Contrastingly, a collectivist culture, such as China, had a more singular cultural group and did not rely on global immigration during its formation, which would result in interdependent beliefs among individuals. Furthermore, in collectivist cultures power imbalances are common, as there is a greater respect toward authority and acceptance of the imbalances, because the population focuses on the greater benefits provided to all [42,43]. Comparatively, individualistic cultures have more equal power balances, as individuals feel less obligated to conform within their culture [44]. 

Much of the earlier research on collectivism and individualism hinged on how people’s social roles or interactions differed, with modern research indicating that emotions, motivation, and cognition can also differ between the two categories [45]. Initial evidence also suggests that individualism-collectivism differences modified higher-level processes of decision-making by showing that individualists are more rational and collectivists are more interdependent when making decisions [46]. However, a drawback of the individualism and collectivism classification is its inherent focus on how a person perceives her/his personal and societal relationships or motivations, which can be seen as a portion of analysis-holism cognitive perception [45]. 

#### 2.1.3. Convergent Versus Divergent Style

An alternative way to differentiate cognitive styles is by considering convergent and divergent thinking [47,48]. The employment of these cognitive styles has provided researchers a dimension and pathway to understand how people process information. A convergent thinking style can be interpreted as individuals converging on the “correct” answer to a problem by focusing on a singular aspect of a situation [49]. Oppositely, divergent thinking is associated with generating multiple solutions, considering wide varieties of information, and focusing on many portions of a situation or problem [50,51]. Divergent thinking has been linked to creativity and this linkage has been further supported by neural imaging research indicating similar areas of brain activity between divergent and creative thinking styles [52,53]. Cognitive styles are rarely binary, and these findings reflect how consumers can be affected by many factors in how they process information and are divided on the convergent-divergent spectrum. 

Related to divergent thinking are traits paralleling creative tendencies, such as openness to experience, extraversion, imagination, and curiosity [54,55]. Convergent thinking individuals will tend to have negative emotions and divergent thinking individuals more positive emotions [56]. With divergent-convergent cognitive styles capable of altering personality and emotions, one would predict that higher-order neural processes (i.e., decisions or learning) may also be affected. Evidence suggests that decision-making, under convergent versus divergent thinking, utilizes contrasting information, while also activating opposing neural pathways [57,58]. Consequently, individuals on the opposite ends of the divergent-convergent continuum may likely reach differing decisions. Like analytic versus holistic categorization, convergent-divergent thinking has also been shown to differ between individuals from Eastern and Western countries, and additional insight has detailed how it can be seen as a sub-portion of analytic-holistic thinking [25].

#### 2.1.4. Left Brain Versus Right Brain Style

One aspect of cognition that is sometimes underutilized is explaining how cognitive categories may relate to actual areas of the brain, or the hemispherical lateralization concept (HLC). Initial research in the 1960s and early 1970s suggested that humans have two “halves” of the brain by studying patients undergoing procedures to disconnect the corpus collosum [59,60]. However, even as early as the 1970s, these views that the halves of the brain work independently and the possibility of classifying people as “right” or “left” brained were challenged [61]. Findings further led to discussions about how areas within the left hemisphere (i.e., verbal, motor skill, and analytic logic processing) interacted more with areas in same hemisphere, and right hemisphere regions (i.e., spatial reasoning and creative processing) interacted across both hemispheres [62]. Updated HLC research discusses how there are proven relative differences between the lateralized halves of the brain [63,64]. However, these relative differences are not dichotomous between left or right brained people; rather, they are bookends on a continuum where people may lean when processing information. 

As research elucidated that brain lateralization was not binarily right or left brain, a focus on brain asymmetry provided insight into how HLC can categorize individuals. Studies specifically aimed to distinguish how falling onto different portions of the HLC spectrum may influence behavior via contrasting neural pathways [65,66]. These hemispheric contrasts in brain activity go beyond subconscious functions and indicate that behavioral differences can be imparted by relative activity variations between the left and right brain [64,67]. Education researchers have detailed how cognitive differences may exist between individuals tending to employ more right (intuitive or creative) or left (logical or analytic) brain activity when learning and problem solving [68,69]. 

One related way to conceptualize cognitive style is the relational-experiential or cognitive-experiential self-theory (CEST). This cognitive theory categorizes individuals along a spectrum from analytic/rational, associated with deliberate and logical thought, to intuitive/experiential, associated with intuition and emotionally driven thought [70]. One major difference resides in HLC focusing on geometric cerebral guidelines that mediate the cognitive styles and CEST focusing on the behavioral outputs. Another differentiating factor of CEST is that it classifies individuals on type of thought (automatic versus deliberate) rather than on actual cognitive styles [70,71]. Within the analytic-holistic theory, the behavioral tendencies of CEST and HLC are both seen, without the limiting factors of different thought types or the debate on the theoretical accuracy and validity of hemispheric lateralization [64,72]. A consistent theme across cognitive style classifications is the focus on a specific portion of an individual’s information processing or decision-making tendencies. These categorizations fit under the encompassing umbrella of analytic-holistic cognition, which does not rely on niche aspects of information processing or problem solving [13,25]. 

### 2.2. Influences of Cognitive Style on Perception and Liking of Sensory Stimuli

#### 2.2.1. Single Module Stimuli 

Much of the cognitive style research has pertained to psychological studies focusing on theory and problem solving. There is a lack of research connecting cognitive style to how various types of stimuli are processed between individuals with contrasting thinking styles. To begin to understand how cognitive styles may induce changes in stimuli perception, it is first important to clarify single module and multi-module stimuli. A single mode stimulus is one that is directed toward one sense (i.e., a single basic taste, the color blue, or a single olfactory stimulus), while a multi-mode stimulus is one that involves many stimuli across the senses and within the same sense (i.e., a cheeseburger using sight, olfaction, and gustation, or a movie involving multiple visual and sound stimuli). Research suggests that contrasting cognitive styles may be associated with changes in how individuals process simple versus multi-module stimuli [73,74]. 

Single mode stimuli can be connected to each of the five senses: olfaction, visual, gustatory, auditory, and tactile (touch) cues. Research specific to olfaction indicates potential impacts of cognitive style on olfactory perception, with some researchers predicting that cognitive style may be a mediating factor of perceptual differences or be important in olfactory perception in conjunction with non-cognitive factors [75,76,77,78]. Visual stimuli perception displays similar trends, with researchers elucidating how neuroimaging results of individuals support the notion of thinking style altering the interpretation of visual cues [79,80]. Relative to olfactory and visual cues, gustatory cues have received very little specific attention in research. However, synesthesia (i.e., one stimuli triggers interpretation using another sense) involving gustation can be linked to cognitive style according to initial studies on the subject [81]. Auditory processing among individuals has received some academic and industry attention with respect to cognitive style, as music perception and preference were found to be associated with cognitive style differences [82]. Tactile cues have also received some attention in association with cognitive style differences, and findings indicate tactile responses and interpretation partially depended on the individual’s cognitive style [83,84]. 

#### 2.2.2. Multi-Module Stimuli

Cross-modal correspondences and synergistic effects can be seen across multiple senses, and they involve multi-module stimuli, such as food or drinks, and are more representative of consumer’s daily perceptions [85]. Cross-modal correspondences are an example of multi-module stimuli, meaning that they are dependent on a stimulus engaging multiple senses. Multi-module stimuli are increasingly representative of realistic situations, as the brain rarely receives unimodal stimuli [86]. These multi-module stimuli are used to support the concept of multisensory integration, which has garnered attention in explaining how humans process and interpret their surroundings by employing a flexible, combinative neural network [87,88]. Recent models measuring the multisensory integration have suggested methodologies that are more accurate in capturing human responses to multi-module stimuli when considering a compilation of all stimuli across multiple senses [89]. 

A multitude of researchers agree that the human-food interaction is a multi-module experience in which consumers rarely separate individual senses independently [90,91,92,93]. Interactions among the senses have been known to exist, with newer research beginning to detail additional cognitive interactions among the senses that are capable of influencing the neural processing of multiple food stimuli [94]. An important part of consumer perception of food is neurological and psychological processing of the food-related stimuli to reach decisions. Consumer cognitive style has proven to be an effective variable in explaining how food-related opinions and decisions are formulated. For example, Hidalgo-Baz et al. [95] discussed how an individual’s cognitive style could impact how she/he perceived the quality of organic and conventional foods after receiving information about and interacting with products, with parallel results found in relation to processing depth of food stimuli [96]. Jeong and Lee [97] furthered these arguments by discussing, over a variety of food and beverage samples and situations, that the cultures associated with differing cognitive styles consistently display different perceptions of the food or beverage. Differences in how consumers process food-specific information cause ensuing schisms in perception and liking, which have been shown to be dependent on the individual’s cognitive style.

## 3. Influences of Analytic or Holistic Cognitive Style on Food Perception and Eating Behavior

### 3.1. Single Module Stimuli 

Due to its more general and encompassing nature, analytic versus holistic thinking provides potential to investigate how the analytic-holistic cognitive style can predict and explain consumers’ food-related processing, decision-making, and consumption behaviors. Within the research regarding the effects of cognitive style on information processing and stimuli perception, there is some debate on the significant differences being due to contrasting cognitive styles between groups [79,98]. A lack of consistent findings suggests differing methodologies, which has been common when cognitive styles are investigated. To prevent research on consumer thinking style becoming too narrow and missing relevant data, the analytic-holistic cognitive style classification is subsequently suggested. When developing the framework of the analytic-holistic theory and the AHS, a main goal was to ensure that the final result was a general classification style representative of the cognitive steps individuals undergo when processing information [13,32]. Considering analytic-holistic classification when investigating the effect of cognitive styles on consumer perception is therefore predicted to produce more consistent results and more accurate predictability. 

This classification style, however, is only recently developed and has been validated with relatively few studies on how analytic-holistic cognitive style can impact single-module stimuli compared to other cognitive style theories. In addition to analytic-holistic cognition being a novel classification tool, researchers within the field have often suggested it for identifying behavioral, decision, and cultural differences [29,38]. Directing methodologies and studies toward higher-level processing to understand contextual and realistic decision-making has left a gap in the research toward singular areas of stimuli perception. The most studied single-module stimuli type has been visual, with researchers consistently finding that analytic (or holistic) people consider less (or more) contextual information related to visual stimuli [24,25,99]. Cultural neuroscience has found that analytic and holistic individuals differ in their neural development, functioning of higher-order informational processing regions, and emotional processing [100,101]. From the neuroscience findings, it would thus be expected for analytic and holistic individuals to differ in their perception of stimuli in each of the five senses. Table 1 details specific examples of researchers finding analytic-holistic differences in single-modal stimuli. If even basic stimuli can elicit such responses, multi-modal stimuli may result in further response differences between cognitive groups. In addition, considering multiple modes of stimuli can address aspects of ecological validity [102], and virtual reality sensory testing [103] that emphasizes the need to test variables, such as cognitive style, in realistic settings for consumers.

### 3.2. Multi-Module Stimuli

Multimodal sensory experiences induce more complex, interactive stimuli that the brain responds to and interprets into a single cohesive experience, and thus a multitude of shared brain areas associated with the senses are involved in food and beverage perception [113]. In most foods one would consume, there are many interacting stimuli depending on each food matrix. Humans are poor at separating each of these components to be interpreted monadically; rather, they are experienced together to allow for a comprehensive perception and hedonic impression of the food [93,114]. To answer the type of question of how food may be perceived differently between consumers, some research has discussed how multi-module information and stimuli can be perceived inversely between analytic and holistic cultures [115]. 

Studies applying the factor of analytic-holistic cognitive styles to advertising have found that an explanation for the differences in perception of and responses to food advertising can be offered by accounting for the cognitive tendencies of consumers [108]. Holistic thinkers were more sensitive to food advertising claims made by the researchers and their opinions of the food products were more variable depending on the type of advertisement shown. These findings parallel Nisbett et al. ’s [13] description of holistic individuals being more likely to consider contextual information. Such results could apply to environmental cues or packaging claims being more effective with holistic consumers. An interesting application of analytic and holistic differences adjacently related to food-related stimuli involves the findings from Santos et al. [112]; they detailed that analytic and holistic groups differed when they handled contradicting information. As it is well known that emotions and emotional processing significantly affect food perception [116], a logical application of such findings suggests that analytic and holistic individuals would also have contrasting food perceptions due to the inequivalent process in which those from the cognitive groups utilize and apply emotions.

### 3.3. Eating Behavior

A perplexing issue to food and sensory professionals is the complexity and lack of common understanding of consumer food behavior areas, such as food consumption or selection behaviors [92,117]. Some researchers tend to become hyper-specific when understanding aspects of consumer behavior toward foods by applying existing theories, while others tend to take a more general, exploratory approach [118]. Researchers found that consumer behavior toward food is not consistent across cultures when they took a step back to look at the exploratory and comparative picture of their results [119]. An aspect of food-related consumer experiences often receiving attention is eating behavior and understanding how or why eating decisions are made. Sensory researchers in both industry and academia aim to understand eating behavior to effectively market foods to match eating behavior and address health issues [120]. To better explain some of the unknowns of eating behavior, researchers have begun to combine the extrinsic and personal factors by investigating the potential role of cognitive style in guiding eating behavior. Results investigating eating behaviors of specific cultures help support the notion of analytic-holistic cognition being an influencing variable on eating behavior [121]. Another point made about the analytic-holistic cognitive style contrasts between cultures is that the differences can also exist within a culture [122]. Adding analytic-holistic cognition as a variable in analyses has the potential to explain unexpected findings or clarify results by separating two previously unseen groups within a single population. 

Prior studies delineated how decision-making differs between analytic and holistic groups, most notably in terms of the amount and type of information considered for the decisions [13,24,25,123]. Researchers built upon those findings and applied them to food-related conditions, where it was found that the analytic-holistic theory could help explain food product advertising and purchase behavior differences [106,124,125]. These food specific findings match with previous research indicating that holistic consumers should see more contextual relationships between sensory cues. A collective summary of the research connecting and supporting analytic-holistic cognitive styles in sensory and consumer science-related areas is provided in Table 1. Cognitive styles in general are important in these applications, and the analytic-holistic cognitive theory offers an advantage because of its encompassing nature and incorporation of past cognitive theories during its development. 

## 4. Future Directions

Through the explorations of how cognitive style theories, especially the analytic-holistic theory, can offer meaningful insights and increased ability to predict consumers’ reactions to food-related stimuli, a multitude of pathways for future investigations can be considered. One path involves identifying how analytic and holistic food-related differences can translate to traditional cross-cultural comparisons. Much of these cross-cultural comparisons have relied on geographical boundaries to separate cultures, such as analytic and holistic groups, respectively, being associated with Western and Eastern countries. Beekman and Seo [36,110] detailed such analytic-holistic differences, but as their sampling was within an area that would historically be treated as a singular culture (i.e., Northwest Arkansas), these findings speak to the validation of within-cultural analytic-holistic differences found by earlier researchers [32,122]. Such food-related analytic-holistic differences would be replicable in studies investigating more traditional geographically based cultural comparisons, as has been shown repeatedly with the analytic-holistic theory [25]. Another point to consider is to ensure a wider distribution of sampled populations outside of wealthy, developed nations (i.e., WEIRD countries), as researchers have discussed that findings from these populations might not be universal [126,127]. The incorporation of food-related applications of the analytic-holistic cognitive style theory offers an additional factor to the concept of cross-cultural comparisons. Interestingly, it is well known that within a country, there is often a multitude of smaller, distinct food cultures and accepted food norms [125]. Since cultures often involve a deep connection with food, a potential interaction of geographical culture (i.e., country) and local food culture could exist when considering cross-cultural research. Therefore, it would be worthwhile to investigate how the inclusion of analytic-holistic theory can produce translatable findings across traditional cultures rooted in country borders, and more regional cultures that individuals identify with that have distinct food traditions. 

Another area of future work that offers promise is to consider how specific aspects of the analytic-holistic theory may be more applicable to certain areas of sensory perception and consumer behavior. For example, Brauch and Größler [128] recently found that in the applied area of systems management, only the analytic-holistic construct of causality was a significant predictive factor. An interesting application here would be employing the analytic-holistic theory to explore its potential mediating impacts on the expansive findings from the Italian Taste project [129]. Consequently, the different areas of the food experience outlined by Beekman and Seo [110], i.e., food shopping, preparing, and consuming, may be impacted differently by individual analytic-holistic constructs. Such a notion is supported by earlier discussion from Miyamoto [38] that detailed how the analytic-holistic theory was developed to be a more general, encompassing theory, and thus such general cognitive tendencies may be modulated in applied scenarios. A third area that would build upon this research is to test the consistency of analytic-holistic differences in food-related scenarios across a wider range of samples and sensory tasks and scenarios. As consumers do not perceive all types of food and beverage samples equally, it is crucial to widen the spread of types of samples this theory has been validated with. Related, sensory evaluation can involve a broad and ever-expanding array of tasks to gauge consumer perception. It is necessary to understand how analytic-holistic differences may manifest across these various sensory evaluation tasks, as each of them can involve consumers undergoing slightly different cognitive processes that may modulate the analytic-holistic effects 

An additional area of interest that extends research findings of food, sensory, and cultural psychology research is investigating the effect of time spent within a culture on the analytic-holistic cognitive style. This exploration can also be expanded to identify how differing time lengths in various cultures can affect one’s food perception, food consumption, or food choice habits. Tan et al. [130] were able to show that small exposures of individuals from an analytic culture to a more holistic culture affected individuals’ physiological states, neural patterns, and creativity. Thus, if these parameters were all influenced by multicultural experiences of less than an hour, it would be expected that food perception and behavior would also be impacted, especially given that most multicultural experiences are much longer than one hour. This hypothesis is supported by adjacent research discussing how longer-term exposure to differing cultures can affect the tendency to adopt that culture’s style of thinking [131]. With increased globalization and the sharing of cultures, it may also be fruitful to consider the exposure with other cultures, which may also moderate the results. However, other researchers have brought up the fact that the time of association with a culture is not a mutually exclusive variable in how an individual can adopt the culture’s associated cognitive style. Through horizontal cultural transmission (i.e., social media) the traits of individualism are more easily transferred, while through vertical cultural transmission (i.e., family contact and values) the traits of collectivism and situational attribution are more easily transferred [132]. These cognitive traits are portions of the analytic-holistic theory, meaning that the other main portions of the analytic-holistic theory would also be expected to parallel these findings. Furthermore, a study by Na et al. [133] found that expected cultural-cognitive differences between analytic and holistic cultural participants were more pronounced with older individuals. This finding therefore suggests that a longer amount of time spent within a single culture may strengthen the associated cognitive tendencies of that culture. If there is an age, culture, and family interaction of how time spent in different cultures affects cognitive style changes, both time and family dynamics along with exposure are salient variables for further consideration. As consumer perception and behavior toward foods are known to be increasingly complex, it is crucial to account for these variables to try to explain even a small portion of the variability in the search to understand the consumer-food interaction. Following along these paths of future investigation can offer researchers a clearer understanding of the significant impacts of consumers’ cognitive style on food perception. 

## 5. Conclusions

Throughout the twentieth century, many psychology researchers classified individuals into cognitive styles to better understand human differences in perception and behavior. Many of the earlier cognitive style theories involved niche applications or areas of cognition to separate individuals. Thus, even though these theories offered great insights, they were limited in their scope and overall generalizability. In the early years of the twenty-first century, the analytic-holistic cognitive style theory was developed to provide a more general, all-encompassing theory to separate individuals based on their cognitive tendencies. As cognitive style research hinges on the notion that all people do not perceive and process stimuli in the same fashion, these theories have great applicability to the field of sensory and consumer sciences. One of the paramount research questions within sensory science is to better understand how and why consumers perceive and respond to food stimuli in the ways they do. Applying cognitive style theories, especially the analytic-holistic theory, offers great promise for better understanding consumers’ food perception and behavior. Initial research has found that analytic and holistic consumer groups do significantly differ in how they perceive and respond to food stimuli. These findings have opened the door to help researchers better understand one of the most complex aspects of food science: the consumer.

## Figures and Tables

**Table 1:** Summary of peer-reviewed articles related to the analytic-holistic cognitive theory and its associated cultures with respect to consumer perception and behavior in food-related contexts.

Publications	Method to Separating Participants/Populations of Comparison	Applicability to Sensory and Consumer Sciences	Main Findings
Chrea et al. [104]	Separated cultures by country comparing French, American, and Vietnamese participant groups	Compared how different cultural groups evaluated and sorted olfactory stimuli	French and American groups differed in olfactory evaluation compared to Vietnamese group
Zhang & Seo [5]	Separated groups by cultures through comparing Chinese and American participants	Compared attention given to portions of plates of food between the two cultures	The Chinese group provided more attention to the context of the food plates while the American group provided more attention to the food items
Bakhchina et al. [105]	Comparison within Russian population using AHS to separate groups	Compared heart rate and visual response times for tasks regarding object-field relation	The analytic group had longer visual response times and higher heart rate when evaluating objects in relation to the field than when evaluating objects irrespective to the field
Hildebrand et al. [106]	Separated participants using the AHS	Investigated how analytic and holistic groups differ in their self-control of indulgent food advertisements with occasion-setting components	The holistic group had higher cravings and purchase likelihood for indulgent samples when shown advertisements with context cues compared to the analytic group
Togawa et al. [107]	Comparison within Japanese population using AHS to separate groups	Investigated the crossmodal correspondence of visual and gustatory senses in product packaging	The holistic group was more affected by the visual-gustatory crossmodal correspondence than the analytic group
Yang et al. [108]	Employed AHS-based priming procedure to induce analytic vs. holistic thinking	Focused on how brand marketing strategies impact consumer response and perception	The holistic group formed more positive responses when shown moderate advertising strategies while the analytic group was relatively unaffected
Peng-Li et al. [109]	Separated groups by cultures through comparing Chinese and Danish participants	Investigated the crossmodal correspondence of sound and basic tastes between the two cultures	The Danish group gave more attention to the food samples while the Chinese group gave more attention to the context of the food dish
Beekman & Seo [110]	Separated participants from Northwest Arkansas using the AHS	Identified how the analytic-holistic theory can apply to consumer food experience	Findings show the analytic-holistic theory applies throughout the consumer food experience with cognitive group differences in line with prior psychological work
Gupta et al. [111]	Separated groups within an Australian population into Western and Eastern cultures	Compared if sensory evaluation and measurement tools could differentiate cultural groups’ food ratings	CATA emotions, CATA emojis, and facial expression analyses could differentiate cultural groups but hedonic ratings could not
Santos et al. [112]	Separated participants from the USA using the AHS	Compared responses to contradictory food-related information	The holistic group was more accepting of contradictory information than the analytic group, and this was in part managed by a higher degree of mixed emotions
Beekman & Seo [36]	Separated participants from Northwest Arkansas using the AHS	Compared the environmental eating effect on food perception between analytic and holistic groups	Compared to the analytic group, the holistic group was more impacted by the eating environment

AHS: Analysis-Holism Scale; CATA: check-all-that-apply.

## Data Availability

Not applicable.
